# Microbial Quality and Production Methods of Traditional Fermented Cheeses in Lebanon

**DOI:** 10.1155/ijfo/5673559

**Published:** 2025-01-08

**Authors:** Mabelle Chedid, Houssam Shaib, Lina Jaber, Youmna El Iskandarani, Shady Kamal Hamadeh

**Affiliations:** ^1^The Livestock Sustainability (TLS), Montpellier, France; ^2^Department of Agriculture, Faculty of Agricultural and Food Sciences, American University of Beirut, Beirut, Lebanon; ^3^The Environment and Sustainable Development Unit, Faculty of Agricultural and Food Sciences, American University of Beirut, Beirut, Lebanon

**Keywords:** Ambarees, fermented cheese, goat milk value chain, Kishk, Lebanon, traditional cheese

## Abstract

This study is aimed at evaluating the quality and safety of two traditional fermented dairy products commonly found in Lebanon (Ambarees and Kishk in its dry and wet forms) by detecting foodborne pathogens and indicator microorganisms. Additionally, it seeks to identify the strengths, weaknesses, opportunities, and threats to quality and the production level. A total of 58 random samples (duplicated) including goat milk (*n* = 16), dry Kishk (*n* = 8), wet Kishk (*n* = 8), and Ambarees (*n* = 26) were collected from individuals who both farm and process these products. They represent the two production levels within the identified value chains in the Shouf and West Bekaa regions. Microbiological analyses revealed that all samples were free of *Escherichia coli*, while *Staphylococcus aureus* was found in all of them with significantly higher levels of contamination in wet *Kishk*, indicating poor hygienic practices at the different production levels. Coliforms were found in only 3.8% of the *Ambarees* samples, made from raw milk, showing that the decreasing pH and water activity throughout the long milk fermentation process contributes to the low prevalence of these microorganisms and that cross-contamination might have occurred during the packaging phase. A regional pattern was observed where microbial counts (total aerobic counts, total coliforms, and *S. aureus*) were found significantly higher (*p* < 0.05) in milk samples of the West Bekaa, suggesting better hygienic conditions in the Shouf as compared to the West Bekaa. This analysis would be an important tool for developing the goat dairy sector and enhancing the production and promotion of traditional goat milk products. Valorization at the different levels of the value chain, starting with the adoption of good production practices on the farm, training on hygienic standards, innovation in packaging and marketing, and developing adequate policies, may ensure high-quality end-products that will eventually contribute to the livelihoods of the main stakeholders in this value chain and help preserve these national culinary gems.

## 1. Introduction

Small ruminant production, specifically goat farming, has become an agricultural activity of high importance worldwide, and not only in countries with low income [1]. Demand for goat dairy products is rising in both traditional and new markets. Goat milk and milk-based products are often preferred for their health and nutritional benefits, including greater digestibility and lipid metabolism, in addition to their distinguished taste, as compared to cow milk [2]. According to the Food and Agriculture Organization of the United Nations [3], the global goat population continues to grow, reaching more than one billion heads in the world. In 2017, the global dairy goat population was estimated to be 218 million [4], and the number of goats raised primarily for milk production keeps on rising. This number increases, especially in low-income countries, due to the high ability of goats to survive in harsh environments and hence are produced in the most marginal regions of the world. Caprine production value chain contributes to the livelihoods of a large number of farmers globally, including in the Mediterranean and the Middle East [1, 5], thus playing an integral part in the livelihoods of particularly poor farmers [4, 5]. The goat milk sector in Lebanon continues to improve despite many challenges faced at the farm (lack of finance, declining interest of youth, etc.), national (lack of government support, market, and struggling local economy), regional (Syrian crisis), and global levels (climate change) [6]. Indeed, goat milk production has increased from 21.2 thousand tons in 2008 to 34 thousand tons in 2010 [7], and Lebanon continues to be one of the highest per capita consumers of dairy products globally (189 L equivalent milk per capita) [8].

Goat milk undergoes various traditional fermentation processes, resulting in common products like yogurt and cheeses that are prevalent nationwide. Meanwhile, there are also region-specific options such as Ambarees in the Northern and West Bekaa, also known as Serdalli in the Shouf area [9]. The production of these region-specific products remains rooted in traditional methods, passed down through generations and retaining their artisanal character. In contrast, the production of more widespread items has largely transitioned to a commercial scale, now carried out by both small and large dairy operations. However, the production of these dairy products suffers from several constraints at different levels of their value chain, from the low productivity of the herd [10] to the poor management and hygiene at both the farm and processing units [9] and the improper packaging and inefficient distribution networks [11]. As a result, goat milk products may be unappreciated and lack consumers' trust and good marketability.

This study is aimed at assessing the quality and safety of traditional fermented goat dairy products in two pastoral areas of Lebanon: the Shouf and West Bekaa, as a first step to identifying possible interventions to improve the quality of these products and develop their related value chain. This analysis would be an important tool for developing the dairy sector and enhancing the production and promotion of goat milk products, which may eventually contribute to the livelihoods of the stakeholders of this value chain, including the goat farmers and processors.

## 2. Materials and Methods

### 2.1. Description of the Study Area: The West Bekaa and Shouf

With a surface area of only 10,452 km^2^, Lebanon is one of the smallest countries in the Middle East and the Mediterranean region. Its typical Mediterranean climate is characterized by heavy rains during the winter season (November to May) and dry months through the rest of the year. This varied range of microclimates leads to a big diversity in vegetation and agricultural products. The Bekaa Valley is the largest agricultural part of the country, comprising three zones with different livestock production systems. It is famous for its fertile agricultural lands, especially in its southern part known as the West Bekaa, which hosts large numbers of small ruminants estimated to be 80,116 (38.8% of the Bekaa goat herd) and 40,364 (21.2% of the Bekaa sheep herd) heads of local *baladi* goats and fat-tailed *Awassi* sheep, respectively [7], both highly adapted to the semiarid conditions prevailing in the region [12]. The Shouf area, which is located in the southern part of Mount Lebanon, is separated from the West Bekaa by the *Jabal el Barouk* Mountains. The Shouf is characterized by small-scale agricultural lands, dairy farms, and short milk value chains, which contribute significantly to the economy of the area [13]. In both regions, goats are reared for milk production, including typical products like *Ambarees*, *Kishk*, and *Labneh* balls preserved in olive oil [14]. The presence of the Shouf Biosphere Reserve (SBR), which stretches over 24 villages in the Shouf and West Bekaa and which yearly attracts thousands of local and international tourists, increases the areas' potential for food-tourism projects that are likely to highlight the local goat milk production systems and hence contribute significantly to rural livelihoods.

### 2.2. The Production Process of Ambarees, Dry Kishk, and Wet Kishk

#### 2.2.1. Ambarees

*Ambarees*, also known as *Serdalli*, is a Lebanese dairy delicacy with a long history, which is produced in some villages of the Bekaa Valley and the Shouf Mountains. The process of *Ambarees* production makes it a climate-smart product where seasonal goat milk is transformed into a pungent cheese preserved for use during winter when goat milk is not available. Traditionally, the preparation process starts towards the end of June when goats are grazing on dry herbs with low moisture content and is carried on until autumn [15]. Raw milk is naturally fermented in an earthenware jar, replaced today with plastic barrels, with continuous draining of whey from a hole in the lower part of the jar and the addition of raw milk and coarse salt until the vessel is full of *Ambarees* curd. After three to four cycles of adding new milk, the jar is sealed, and the milk completes its fermentation up to 4–6 months before the jar is opened [9]. According to interviews conducted with Ambarees producers, the collected milk must be gathered from the same herd during the milking season to ensure the quality of the product. Towards November and December, the *Ambarees* is moved from the jar to cloth bags for further drying for around 48 h. Once dried, *Ambarees* could be used in making *Kishk* or simply preserved in glass jars and covered with olive oil. Vacuuming *Ambarees* in plastic bags has become a modern practice nowadays. Making *Ambarees* has become a rare practice found only in remote areas of rural Lebanon due to a lack of technical know-how and a lack of specially made jars, which made this typical cheese a sought-after product.

#### 2.2.2. Dry Kishk and Wet Kishk

*Kishk*, on the other side, is a fermented dairy product known across different cuisines of the Middle East (Iran, Iraq, Turkey, and Lebanon) and India [16]. In Lebanon, *Kishk* is prepared by fermenting cracked wheat (*bulgur*) in milk or yogurt. Cow, sheep, or goat milk is used [17, 18]; however, goat milk gives its typical acidic taste, while cow milk is milder and mainly used to meet the taste of urban consumers. *Kishk* is prepared during summer to serve as a provision for wintertime. The low moisture content and acidic pH of the final product make it safe against the growth of pathogenic microorganisms [19].

To produce *Kishk*, coarse bulgur is soaked in yogurt in a large clay bowl, and the mixture is kneaded daily for 4 days until the yogurt is absorbed. During this time, yogurt is added on a daily basis to prevent the *Kishk* from drying, and salt is also added to prevent mold growth [17]. After 9 days of fermentation, “green” or wet *Kishk* is obtained. This by-product is either consumed fresh or preserved in olive oil. The remaining wet *Kishk* is then formed into balls and sundried on clean sheets on the roof while being rubbed every day in order to fasten the drying process. When Kishk completely dries, it is ground into a fine powder and then stored either in jars or cloth bags [17, 20].

### 2.3. Sampling Methods

The study targeted different regions in Lebanon, with two main ones recognized for Ambarees and Kishk production: Shouf and West Bekaa. Within these two regions, Dimassi et al. [21] have identified 39 active small-scale producers, 27 in West Beqaa and 12 in Shouf. Accordingly, in the current work, the same producers were contacted, with 36 who responded and were consequently included in the study, 26 from West Beqaa and 10 from the Chouf.

Retrospective sampling methods were employed during the collection process. Samples, each weighing 50 grams, were gathered using sterile cups during four distinct visits to the sampling locations. A total of 58 samples were gathered through duplicate testing. The samples encompassed goat milk (*n* = 16), Ambarees (*n* = 26), dry Kishk (*n* = 8), and wet Kishk (*n* = 8), sourced from the 36 farmers identified above, engaged in the production of such traditional cheeses within their household facilities for commercial sale. The batches were promptly transported on the same day of collection to the Animal Physiology and Pathology Laboratory at the American University of Beirut under refrigeration at around 10°C for analysis. The primary objective was to evaluate their hygienic quality and to determine the presence of pathogenic and indicator bacteria that are relevant to food hygiene practices. The analysis focused on the enumeration of total aerobic count (TAC), *Escherichia coli*, total coliforms, and *Staphylococcus aureus* to identify any potential association with foodborne illnesses.

### 2.4. Microbiological Analysis

Samples (10 g from each) were aseptically taken and homogenized in 90 mL of sterile diluent (0.1% peptone water) with a Stomacher (Seward, Model 400, England) for 30 s. Seven tube decimal dilutions were prepared using a ratio of 1 mL of the food-homogenized suspension to 9 mL of peptone water [22]. The spread plate method was adopted, whereby a volume of 0.1 mL of each sample dilution was plated onto reference agar, whether general or selective, for the enumeration of various bacteria as per dependable reference methods and guidelines set by the International Dairy Federation (IDF) and the Association of Official Analytical Chemists (AOAC) [21–23]:

1. TAC was determined using Plate Count Agar (PCA) (Bio-Rad, Alfred Nobel Drive, CA 94547, United States). PCA plates were incubated at 37°C for 24 h.
2. The enumeration of *S. aureus* was carried out on RAPID'Staph Agar (Bio-Rad, Alfred Nobel Drive, CA 94547, United States). The medium's principle relies on the ability of *S. aureus* to reduce tellurite (black colonies) and induce egg yolk proteolysis (clear halo around the colony). RAPID'Staph Agar plates were incubated at 37°C for 24 h.
3. The enumeration of total coliform and *E. coli* counts was carried out using RAPID'*E. coli* Agar (Bio-Rad, Alfred Nobel Drive, CA 94547, United States). One set of plates was incubated at 37°C for 24 h to determine the total coliform count, and another set was incubated at 42°C to inhibit the growth of any interfering flora and enhance the specificity of the medium for *E. coli*. The medium's principle is based on detecting the activities of *β*-D-glucuronidase (GLUC) and *β*-D-galactosidase (GAL) simultaneously. When *E. coli* (GAL+/GLUC+) is present, the colonies formed exhibit a violet to pink color, while other coliforms (GAL+/GLUC−) form green colonies.

After incubation, the colonies (30–300 colonies) developed on agar plates were counted and recorded. Each tabulated value represents the mean of duplicates, and results were expressed as colony forming units per gram of sample (CFU/g) [22, 23]. Results were compared to national and international microbiological standards to decide whether the tested products are safe or not for human consumption [24, 25].

### 2.5. Statistical Analysis

Bacterial counts were normalized by Log10 transformation. Means of bacterial counts were compared for statistical significance (*p* = 0.05) using one-way ANOVA and Tukey's test of SPSS (V. 25.0 SPSS, Chicago, IL, United States). The frequency of microbial contamination among samples was compared using the chi-square test, and Pearson's correlation among microbes in each food type was completed using the same statistical software.

## 3. Results and Discussion

### 3.1. Microbiological Characteristics of Collected Samples

The percentage of samples with positive microbial counts is presented in Table 1. While all samples tested positive for TAC, 25% and 3.8% of milk and *Ambarees* samples, respectively, contained coliforms. Although the presence of coliforms has always been associated with fecal contamination, recent studies showed that coliforms are evidence of environmental contamination as well [26]. High levels of coliforms in raw milk (exceeding 1000 CFU/mL, for example) may indicate very poor sanitary practices on the farm, inappropriate refrigeration, or, in some cases, the animals' infection with coliform mastitis [26]. Coliforms do not survive pasteurization. When detected in processed milk or dairy products, coliforms indicate postpasteurization contamination. However, this is not the case for *Kishk* in our study, which was found free of coliforms, similar to the findings of Salameh and Hosri [18], who investigated the hygienic quality of *Kishk* in Lebanon in 2016. The detection of coliforms in only 3.8% of the *Ambarees* samples, made from raw milk, may be an indication that the decreasing pH and water activity throughout the long milk fermentation process contributes to the low prevalence of these microorganisms [27]. Alternatively, cross-contamination could have occurred during the packaging phase.

None of the collected products were contaminated with *E. coli*, whereas all of them tested positive for *S. aureus*, with wet *Kishk* being the most contaminated product (Table 2). The percentage of milk, *Ambarees*, and dry *Kishk* samples contaminated by *S. aureus* was almost the same (61.5%–62.5%), with no significant difference between them (Table 1).

Dry *Kishk* is a highly acidic food characterized by low water activity [28], which may suggest the absence of *E. coli* and other coliforms in this product. However, the obtained results revealed relatively high levels of *S. aureus* in the analyzed *Kishk* samples, hence making this product noncompliant with the national standards [24] and comparable to the *Kishk* samples analyzed by Salameh and Hosri [18], which were heavily contaminated with *S. aureus*, specifically those collected from the South of Lebanon (2.51^∗^10^2^ CFU/g). The same authors also reported relatively high levels of *S. aureus* contamination (3.78^∗^10^1^ CFU/g) in dry *Kishk* samples collected from Bekaa. This observation is most likely indicative of poor production practices, at least at the level of drying *Kishk*, which is a critical phase where *Kishk* is left to dry, uncovered under the sun, and then kneaded with bare hands to remove lumps, and at the level of storing. High levels of *S. aureus* were also detected in all wet *Kishk* samples given the high water activity of this product at this intermediate phase of production. Moreover, the addition of *bulgur* to yogurt while making wet *Kishk* enhances the growth of *S. aureus* by providing additional protein substrate that might increase up to seven points in comparison to yogurt [20, 29]. In addition, the barehanded handling and the open drying process expose the product to multiple external sources of contamination, such as water, soil, animals, and poor personal hygiene. *S. aureus*, which was detected in all products, is the most resistant bacterium to salt and low pH in comparison to the other microorganisms observed in this study. Both *Ambarees* and *Kishk* are acidic and highly salted; hence, the loads of *S. aureus* in such products highlight contamination of raw material used, poor production conditions, and the probability of cross-contamination at the packaging level, which was explained by Dib et al. [30] and Salameh and Hosri [18]. The presence of *S. aureus* in these products is alarming, as this bacterium is closely associated with foodborne illnesses and even the deaths of many people worldwide each year due to acute intoxication with the enterotoxin produced by this bacterium.

### 3.2. Correlations (Total Aerobes, *S. aureus*, and Coliforms)

Correlation between microbial counts (Log10 CFU/g) in milk and in Ambarees samples collected from the same farmer/producer was nearly significant for *S. aureus*, indicating poor hygiene at the level of the animals (milking, bedding, etc.) and at the production level (milk handling, dirty equipment, etc.) (Pearson′s correlation = 0.896; *p* = 0.07). The high acidity of Ambarees did not prevent its contamination with *S. aureus*, which underlines again the ability of *S. aureus* to withstand salinity and low water activity in foods. These results were not in accordance with the findings of Hajj Semaan et al. [9], who reported safe end products collected from the Bekaa except for “Baladi cheese,” which is made of raw milk. The said authors attributed this contamination to a mastitis infection of one goat. No significant correlation was found between coliform count and total count in Ambarees, demonstrating again the vulnerability of these microorganisms vis-à-vis the processing conditions of this fermented food in comparison to other persistent bacteria. Moreover, the fact that a significant positive correlation was also observed between *S. aureus* and TAC in dry and wet *Kishk* samples (Table 3) further underlines the importance of *S. aureus* as a considerable food contaminant that constitutes a major part of the bacterial population in specific food around the world [31].

### 3.3. Regional Patterns and Potential for Improvement

Microbial counts in milk and *Ambarees* showed a regional pattern where microbial counts were found significantly higher (*p* < 0.05) in the West Bekaa for TACs, total coliforms, and *S. aureus* in milk, and for *S. aureus* in Ambarees (Table 4). The obtained results suggest better hygienic conditions in the Shouf as compared to the West Bekaa, across the production chain from milk production [32] to packaging. In fact, upon close analysis of the collected data from the farmers and investigation of the available literature [32–34], two factors were identified with potential impact on the observed difference in microbial quality between the two regions. The first factor is the difference in the material used during processing. Although not all farmers specified this detail, it was noted that earthen jars were used by at least three of the West Bekaa farmers. In contrast, plastic ware was the main material reported by the Shouf producers. Previous studies have reported on the potential role of earthenware in harboring contaminants in traditional dairy products [33, 34]. On a different note, a discrepancy in the water quality between the two regions is highly suspected. Based on the “water supply and wastewater systems master plan for the Bekaa water establishment” study conducted by the United States Agency for International Development (USAID) [32], it was highlighted that the status of the water network in West Bekaa is old and deteriorated, while in Mount Lebanon (including the Shouf) it is in a better state.

Although not tested in this study, water microbiological quality may be a potential contributor to the products' cross-contamination at different stages of processing [35, 36]. As such, ensuring a continuous supply of clean water for dairy producers may be an important step for ensuring quality control and hygiene. Nevertheless, the microbiological figures in the Ambarees produced in West Bekaa are lower than those observed in milk samples collected from the same region. This can be further proof of the favorable impact of *Ambarees* processing conditions on reducing bacterial loads originating from the collected milk.

An additional factor of relevance to product quality is the inconsistent use of cooling and refrigeration in these traditional production methods, partly due to the absence of a continuous electric power supply throughout the country. Consequently, variations in product quality are expected, starting with the raw milk and ending with the packaged product on the shelf ready for consumption. Given the important role of Ambarees production in the livelihoods of pastoral communities, namely, farmers and women, it is essential to improve its quality while preserving its traditional production method and consequently increase its marketing potential, Ambarees being mainly sold at the producers' shelf. The same applies to Kishk, which offers a high potential in rural development and rural women's livelihoods [17]. The role of local products in local development has been long investigated in different communities around the world, and SWOT (strengths, weaknesses, opportunities, and threats) analyses have been done to position these traditional cheeses in the market and propose improvement strategies [37, 38]. Examples of cheeses include the Serbian “Pirotski Kachkaval,” which presents a high potential for “terroir” products [37], and artisanal African cheeses like Wara, Takammart, Wagashi, and Domiati [38]. Traditional and artisanal cheeses owe their uniqueness to their geographical specificity, including human and natural factors, their taste and traditional production methods, their nutritional value, their valorization of local resources, including local livestock breeds, their use of affordable and simple tools, and their low production cost [37–39], which offer a good model to ensure their sustainability [38]. In the case of Kishk and Ambarees, they offer the added feature of climate-smart products with long shelf lives at room temperature (dried Kishk and *Ambarees* in oil), thus making them a valuable year-round, calcium-rich source of nutrition. This feature is an important opportunity for tapping into the potential of these products and improving their value chain when proper quality control is applied. However, artisanal cheeses also have weaknesses, including, among others, an uneven distribution of value through their value chain despite the premium price for producers [13, 37], the lack of producers' associations, inadequate marketing skills, the absence of labeling and branding [17, 37], and above all, the low-quality control of milk and the end product [28]. Additionally, their production faces several economic challenges mainly related to no access to credit facilities [6, 10]. The valorization of traditional dairy products, including Ambarees and Kishk, leads to preserving people's culture and heritage; nevertheless, it is important to keep in mind, as some studies suggest [40], that the income of small-scale producers may not necessarily improve since the value is not evenly distributed among the value chain actors and because the quality upscaling may imply certification costs. Hence, preserving the traditional production aspect and short marketing channels, like on-farm sales, for instance, may contribute to granting small-scale processors a premium price and consequently improve their livings. Valorization can occur at the different levels of production through capacity building on hygienic practices in the farm and the transformation unit [17], national and specific policies and hygiene standards adapted to small-scale producers and compatible with artisanal cheese production [17, 41], and territorial innovations that include technological, organizational, social, and institutional innovation [41].

## 4. Conclusions

Ambarees and Kishk are produced traditionally, which means manufacturing methods are passed from generation to generation without a standard technology, standard regulations, or pasteurization. The samples in this study showed various degrees of contamination with coliforms and *S. aureus*, indicating possible pre- and postprocessing contamination. Contamination decreased significantly in the final dry (Kishk) and high-acidity products (Ambarees), indicating the potential to achieve safe results if basic hygiene and good manufacturing practices (GMP) are implemented, eventually leading to improved marketability. At the macrolevel, the analysis highlighted the need for a strategic, market-driven vision to strengthen the interaction between vertical and horizontal links in the value chain, including technical assistance, institutional support, and quality control. Conducive and efficient policies are also needed at the national level to create an enabling environment throughout the targeted goat dairy value chain, leading to quality products and improved rural livelihoods.

## Figures and Tables

**Table 1:** Percentage of samples with positive microbial counts.

**Type**	**N**	**% of samples**				
		**TAC positive**	**Coliform positive**	***E. coli* positive**	***Staphylococcus aureus* positive**	**Microbial counts above acceptable limits [** 24, 25**]**
Milk	16	100.0	25.0	0.0	62.5	25.0
*Ambarees*	26	100.0	3.8	0.0	61.5	11.5
Dry *Kishk*	8	100.0	0.0	0.0	62.5	12.5
Wet *Kishk*	8	100.0	0.0	0.0	100.0	75.0

*Note:* % = number of positive samples/total number of tested samples (*N*).

Abbreviation: TAC = total aerobic count.

**Table 2:** Microbial counts in various dairy products.

**Type**	**N**		**Microbial count (Log10 CFU/g)**		
		**Total aerobic**	**Total coliform**	***E. coli***	***Staphylococcus aureus***
Milk	16	3.60 ± 1.04^a^	0.65 ± 1.46^a^	0.00 ± 0.00^a^	2.24 ± 1.93^a^
*Ambarees*	26	3.82 ± 1.02^a^	0.09 ± 0.48^a^	0.00 ± 0.00^a^	1.74 ± 1.45^a^
*Dry Kishk*	8	3.93 ± 1.23^a,b^	0.00 ± 0.00^a^	0.00 ± 0.00^a^	1.92 ± 1.76^a^
Wet *Kishk*	8	5.28 ± 1.35^b^	0.00 ± 0.00^a^	0.00 ± 0.00^a^	4.49 ± 0.91^b^
SEM		0.158	0.112	0.000	0.235

*Note:* Means in a column with different alphabetical superscripts are significantly different (*p* < 0.05).

Abbreviations: CFU = colony forming unit, SEM = standard error of mean.

**Table 3:** Correlation between microbial counts (Log10 CFU/g) in dry Kishk (*N* = 8) and wet Kishk (*N* = 8).

	**Total aerobic count**	***Staphylococcus aureus***	**Total coliforms**
Dry *Kishk*			
Total aerobic count	1	0.807^*^	NA
*Staphylococcus aureus*	0.807^*^	1	NA
Total coliforms	NA	NA	1
Wet *Kishk*			
Total aerobic count	1	0.892^*^	NA
*Staphylococcus aureus*	0.892^*^	1	NA
Total coliforms	NA	NA	1

Abbreviation: NA = not applicable.

^*^Significant at *p* < 0.01.

**Table 4:** Microbial counts in milk and Ambarees based on their region of origin∗.

**Type**	**Region**	**N**	**Microbial count (Log10 CFU/g)**		
			**Total aerobic**	**Total coliform**	***S. aureus***
Milk	Shouf	11	2.94 ± 0.46^a^	0.00 ± 0.00^a^	1.34 ± 1.60^a^
	West Bekaa	5	4.95 ± 0.28^b^	2.07 ± 2.08^b^	4.20 ± 0.67^b^
	SEM		0.261	0.365	0.481

*Ambarees*	Shouf	5	4.28 ± 1.24^1^	0.00 ± 0.00^1^	0.60 ± 1.35^1^
	West Bekaa	21	3.74 ± 0.98^1^	0.12 ± 0.55^1^	2.11 ± 1.32^2^
	SEM		0.207	0.098	0.287

*Note:* Means in a column with different alphabetical or numerical superscripts are significantly different (*p* < 0.05).

Abbreviations: CFU = colony forming unit, SEM = standard error of mean.

∗The other dairy products were sampled from the West Bekaa only.

## Data Availability

Data is available upon request from the authors. Requests can be addressed to the corresponding author.

## Nomenclature

CFU g^−1^: coliform forming unit (colony forming units per gram, a representation of the number of bacteria present in 1 g of sample)

SEM: standard error of mean

## References

[1] Dubeuf J. (2011). The social and environmental challenges faced by goat and small livestock local activities: present contribution of research–development and stakes for the future. *Small Ruminant Research*.

[2] Haenlein G. (2004). Goat milk in human nutrition. *Small Ruminant Research*.

[3] Food and Agriculture Organization of the United Nations (2019). *Food and Agriculture Organization of the United Nations Statistical Databases*.

[4] Iñiguez L. Characterization of small ruminant genetic resources in Central Asia, the Caucasus, West Asia and North Africa.

[5] Abdel-Aziz M. (2010). Present status of the world goat populations and their productivity. *Lohmann Information*.

[6] Chedid M., Tourrand J. F., Jaber L., Hamadeh S. (2018). Farmers’ perception to change and adaptation strategies of small ruminant systems in the West Bekaa of Lebanon. *Small Ruminant Research*.

[7] Serhan M., Mattar J., Kukovics S. (2018). The goat dairy sector in Lebanon. *Goat Science*.

[8] Skibiak N. (2014). Small ruminant dairy value chain assessment. *Protect and Provide Livelihoods in Lebanon: Small Ruminant Dairy Value Chain Assessment*.

[9] Hajj Semaan E., Dib H., Abi Ramia R., Chedid M. (2011). Caractéristiques chimiques et qualité bactériologique de produits laitiers caprins traditionnels Libanais. *Lebanese Science Journal*.

[10] Hamadeh S., Shomo F., Nordblom T., Goodchild A., Gintzburger G. (1996). Small ruminant production in Lebanon’s Beka’a valley. *Small Rumin Research*.

[11] El-Balaa R., Marie M. Sustainability of the Lebanese small ruminant dairy products supply chain.

[12] Jaber L., Habre A., Rawda N., Abi Said M., Barbour E., Hamadeh S. H. (2004). The effect of water restriction on certain physiological parameters in Awassi sheep. *Small Ruminant Research*.

[13] Khairallah L., Chedid M., Jaber L. S., Martiniello G., Hamadeh S. K. (2023). Traditional dairy goat value chain in Lebanon: an uneven distribution of values. *Journal of Agribusiness in Developing and Emerging Economies*.

[14] Chedid M. (2019). *Sustainability of Agro-Pastoralist Systems Undergoing Global Changes as Reflected by Farmers’ Perception and Value Chain Analysis: A Lebanese Case-Study*.

[15] Saleh H. (1991). *Microbiological Changes, Chemical Composition and Sensory Properties of Labneh Anbaris Made from Cow's and Goat's Milk-by Hassan Saleh, [M.S. thesis]*.

[16] Kurmann J., Rasic J. L., Kroger M. (1992). *Encyclopedia of Fermented Fresh Milk Products: An International Inventory of Fermented Milk, Cream, Buttermilk, Whey, and Related Products*.

[17] Chedid M., Tawk S., Chalak A., Karam S., Hamadeh S. (2018). The Lebanese Kishk: a traditional dairy product in a changing local food system. *Journal of Food Research*.

[18] Salameh C., Hosri C., Ben Salem H., Boutonnet J. P., López-Francos A., Gabiña D. (2016). Evaluation of the hygienic quality and nutritional value of traditional Lebanese “Kishk”, a dried fermented goat milk product. *Zaragoza: CIHEAM Options Méditerranéennes: Série A. Séminaires Méditerranéens*.

[19] Gadallah M., Hassan M. (2019). Quality properties of Kishk (a dried fermented cereal-milk mixture) prepared from different raw materials. *Journal of the Saudi Society of Agricultural Sciences*.

[20] Tamime A., Barclay M., Amarowicz R., Mcnulty D. (1999). Kishk - a dried fermented milk/cereal mixture. 1 composition of gross components, carbohydrates, organic acids and fatty acids. *Le Lait*.

[21] Dimassi O., Iskandarani Y., Afram M., Akiki R., Rached M. (2020). Production and physicochemical properties of labneh anbaris, a traditional fermented cheese like product, in Lebanon. *International Journal of Environment, Agriculture and Biotechnology*.

[22] IDF (1992). *Milk and Milk Products—Preparation of Samples and Dilutions for Microbiological Examination. Standard 122B*.

[23] AOAC (2005). *Official Methods of Analysis*.

[24] Libnor (2004). *Al-Kishk, Standard No. 250*.

[25] Commission Regulation no 2073/2005 on microbiological criteria for foodstuffs, (European Commission, 2005). https://eur-lex.europa.eu/eli/reg/2005/2073/oj.

[26] Martin N., Trmčić A., Hsieh T., Boor K. J., Wiedmann M. (2016). The evolving role of coliforms as indicators of unhygienic processing conditions in dairy foods. *Frontiers in Microbiology*.

[27] Trmcic A., Chauhan K., Kent D. J. (2016). Coliform detection in cheese is associated with specific cheese characteristics, but no association was found with pathogen detection. *Journal of Dairy Science*.

[28] Serhan M., Mattar J. (2013). Characterization of four Lebanese artisanal goat milk cheeses: darfiyeh, aricheh, shankleesh and serdale by physico-chemical, microbiological and sensory analyses. *Journal of Food Agriculture and Environment*.

[29] Medvedová A., Valík L. (2012). Staphylococcus aureus: characterisation and quantitative growth description in milk and artisanal raw milk cheese production. *Structure and Function of Food Engineering*.

[30] Dib H., Hajj E., Mrad R. (2012). Identification et évaluation de l’effet probiotique des bactéries lactiques isolées dans des fromages caprins traditionnels. *Lebanese Science Journal*.

[31] International Commission on Microbiological Specifications for Foods (1996). *Microorganisms in Foods. 5. Characteristics of Microbial Pathogens*.

[32] Water supply and wastewater systems master plan for the Bekaa water establishment. https://www.pseau.org/outils/ouvrages/dai_kredo_usaid_water_supply_and_wastewater_systems_master_plan_for_the_bekaa_water_water_assessment_report_2015.pdf.

[33] Abi Khalil R., Couderc C., Yvon S. (2022). Artisanal household milk pasteurization is not a determining factor in structuring the microbial communities of labneh ambaris: a pilot study. *Food*.

[34] Bilgin B., Kaptan B. (2016). A study on microbiological and physicochemical properties of homemade and small scale dairy plant buffalo milk yoghurts. *International Journal of Pharmaceutical Research & Allied Sciences*.

[35] Terplan G. (1980). The role of water in the production and processing of milk (author's transl). *Zentralblatt für Bakteriologie, Mikrobiologie und Hygiene. Serie B*.

[36] Burke N., O'Dwyer J., Southern M., Adley C. (2017). An analysis of the microbial quality of water in a milk production plant. *LWT*.

[37] Stojković L., El Bilali H., Berjan S., Despotovic A. Protected geographical indications as a tool for valorising traditional and typical agro-food products and improving rural livelihoods in Serbia: case of “Pirotski kachkaval” cheese from Stara Planina region.

[38] Nyamakwere F., Esposito G., Dzama K., Raffrenato E. (2021). A review of artisanal cheese making: an African perspective. *South African Journal of Animal Science*.

[39] Keskin E., Dağ T. (2020). Identity of cheese: a research on the cheeses of the Aegean region in Turkey. *Journal of Ethnic Food*.

[40] American University of Beirut (2020). Desk review (MedSnail project). https://www.enicbcmed.eu/sites/default/files/2020-11/AUB%2520Desk%2520Review-%2520Final%2520Draft%25201%2520-MedSnail%252030320.pdf.

[41] Pachoud C., Schermer M., Landsteiner E. S. (2019). Reconciling tradition and innovation in traditional mountain cheese value chains: the role of social capital the case of the artisanal Serrano cheese value chain in southern Brazil. *Rural History Year Book: Farming the City*.

